# Social Networking Sites, Depression, and Anxiety: A Systematic Review

**DOI:** 10.2196/mental.5842

**Published:** 2016-11-23

**Authors:** Elizabeth M Seabrook, Margaret L Kern, Nikki S Rickard

**Affiliations:** ^1^ Monash Institute of Cognitive and Clinical Neurosciences School of Psychological Sciences Monash University Clayton Australia; ^2^ Center for Positive Psychology Melbourne School of Graduate Education University of Melbourne Melbourne Australia

**Keywords:** depression, anxiety, social media, social networking, review, systematic, mental health, well-being

## Abstract

**Background:**

Social networking sites (SNSs) have become a pervasive part of modern culture, which may also affect mental health.

**Objective:**

The aim of this systematic review was to identify and summarize research examining depression and anxiety in the context of SNSs. It also aimed to identify studies that complement the assessment of mental illness with measures of well-being and examine moderators and mediators that add to the complexity of this environment.

**Methods:**

A multidatabase search was performed. Papers published between January 2005 and June 2016 relevant to mental illness (depression and anxiety only) were extracted and reviewed.

**Results:**

Positive interactions, social support, and social connectedness on SNSs were consistently related to lower levels of depression and anxiety, whereas negative interaction and social comparisons on SNSs were related to higher levels of depression and anxiety. SNS use related to less loneliness and greater self-esteem and life satisfaction. Findings were mixed for frequency of SNS use and number of SNS friends. Different patterns in the way individuals with depression and individuals with social anxiety engage with SNSs are beginning to emerge.

**Conclusions:**

The systematic review revealed many mixed findings between depression, anxiety, and SNS use. Methodology has predominantly focused on self-report cross-sectional approaches; future research will benefit from leveraging real-time SNS data over time. The evidence suggests that SNS use correlates with mental illness and well-being; however, whether this effect is beneficial or detrimental depends at least partly on the quality of social factors in the SNS environment. Understanding these relationships will lead to better utilization of SNSs in their potential to positively influence mental health.

## Introduction

### Background

Social networking sites (SNSs) are Web-based platforms on which individuals connect with other users to generate and maintain social connections [1]. Considerable disagreement exists as to associations that SNS use may have with depression and anxiety [2,3]. On the one hand, SNSs may protect from mental illness, as they support and enable social interaction and connection [1,4], and allow users to reflect aspects of their identity and express emotion that may be relevant to their lived experience [5]. On the other hand, there are many opportunities for miscommunications and mismanaged expectations, and maladaptive tendencies can be exaggerated, leaving individuals feeling a greater sense of isolation [2,6]. As a whole, the SNS environment may be just as complex as face-to-face interactions. As SNS membership continues to rise [7], it is becoming increasingly important to address the possible benefits and detriments the use of SNSs may have on mental health.

Affective disorders such as depression and anxiety have been shown to have bidirectional interactions with the social environment that influence the path of illness onset and maintenance [8]. Depression and anxiety have an approximate prevalence of 4.7% and 7.3%, respectively, in the global population [9,10]. These disorders have high levels of comorbidity [11] and impact the quality of social relationships [12,13]. Depression and anxiety may be implicated in determining the size and structure of an individual’s social network [12], the quality of interactions within these networks, and how effectively social capital may be leveraged or developed to provide an individual with social support [8,14].

The social characteristics (both qualitative and structural) affected by depression or anxiety are also relevant to one’s sense of well-being. Current mental health theories suggest that the presence of well-being is not the same as the absence of mental illness; a complete model of mental health requires not just the absence of psychopathology, but also a focus on positive indices of functioning such as subjective well-being [15]. This is particularly pertinent when exploring how the social environment may affect an individual, as such environments may simultaneously confer a number of benefits to the individual and exaggerate deficits [16-18].

Social aspects of the Internet have been argued to augment social relationships and support mental health. SNSs in particular connect us to friends, family, colleagues, strangers, and celebrities and can help users to maintain and make new friendships, express thoughts and feelings, and express identity [1,4,19]. The primary social functions that SNSs perform may augment the benefits of engaging in face-to-face interaction by extending the reach and accessibility of our social networks [20]. Indeed, SNS use is associated with lower levels of loneliness and greater feelings of belonging (social connectedness), social capital, and actual and perceived access to social support and is generally associated with higher levels of life satisfaction and self-esteem [6,21-26].

As a whole, the positive social components of SNS use suggest a protective role against depression and anxiety. For instance, higher levels of self-esteem and life satisfaction may aid in attenuating depressive symptoms [27]. Kraut et al [28] found that frequent general Internet use did not increase depression over time, and, in a second study, communication activities on the Internet were shown to be associated with lower levels of depressive symptoms [29]. Computer-mediated communication (CMC; eg, email, instant messaging) allows users to express and interpret emotion in a similar way to face-to-face interaction [17]. CMC may therefore be beneficial for emotion regulation as has been demonstrated for offline forms of written emotional expression [30,31].

However, for individuals with depression or anxiety, the interpretation and frequent exposure to this emotion may have a negative impact [13]. SNS use may increase an individual’s exposure to negative social interactions (eg, cyberbullying), which may negatively impact mood and mental health [2]. For example, negative interaction quality was associated with decreases in self-esteem and life satisfaction [32]. Even passive exposure to the language used in SNS posts has been shown to influence the emotive language subsequently expressed by the receiving SNS user, where positive or negative emotions are argued to transfer via contagion [33-35]. As SNSs explicitly support a number of social features, the relationships and interactions between the user, their emotional experience, and Web-based technology are likely to be complex and may even accentuate differences between those who are doing well in life and those who are struggling.

Cognitive and social factors frequently emerge as both moderators and mediators of the relationships between offline social interactions or events and depression [36-38] and might also occur in Web-based environments. For instance, self-esteem mediates the pathway between relationship interactions and depressive symptoms [39], but it might also moderate how a person uses and is affected by the SNS. Rumination, a response style where an individual maintains a passive and repetitive focus on their distress [40], is one mechanism linking stressful life events and the development or maintenance of depression [41], and the SNS environment provides opportunity for a person to both internally ruminate on bad events and have an entire social network further accentuate shortcomings. Social support has additionally been shown to moderate relationships between stress and depression, with greater levels of social support acting as a buffer to depressive symptoms [42]. This is pertinent to SNSs as they present a potential intervention opportunity for developing and strengthening supportive social networks for vulnerable individuals.

### Objective

Since the advent of SNSs, a number of articles have been published examining the relationship between SNS use and depression and anxiety. The interaction between SNSs and our mental health and well-being is clearly varied and complex. The objective of this paper was to provide a systematic review of literature examining SNSs and their relationship with depression and anxiety. It also considers links with well-being, as well as potential mediators and moderators to these relationships.

## Methods

### Search Strategy

**Figure 1:**
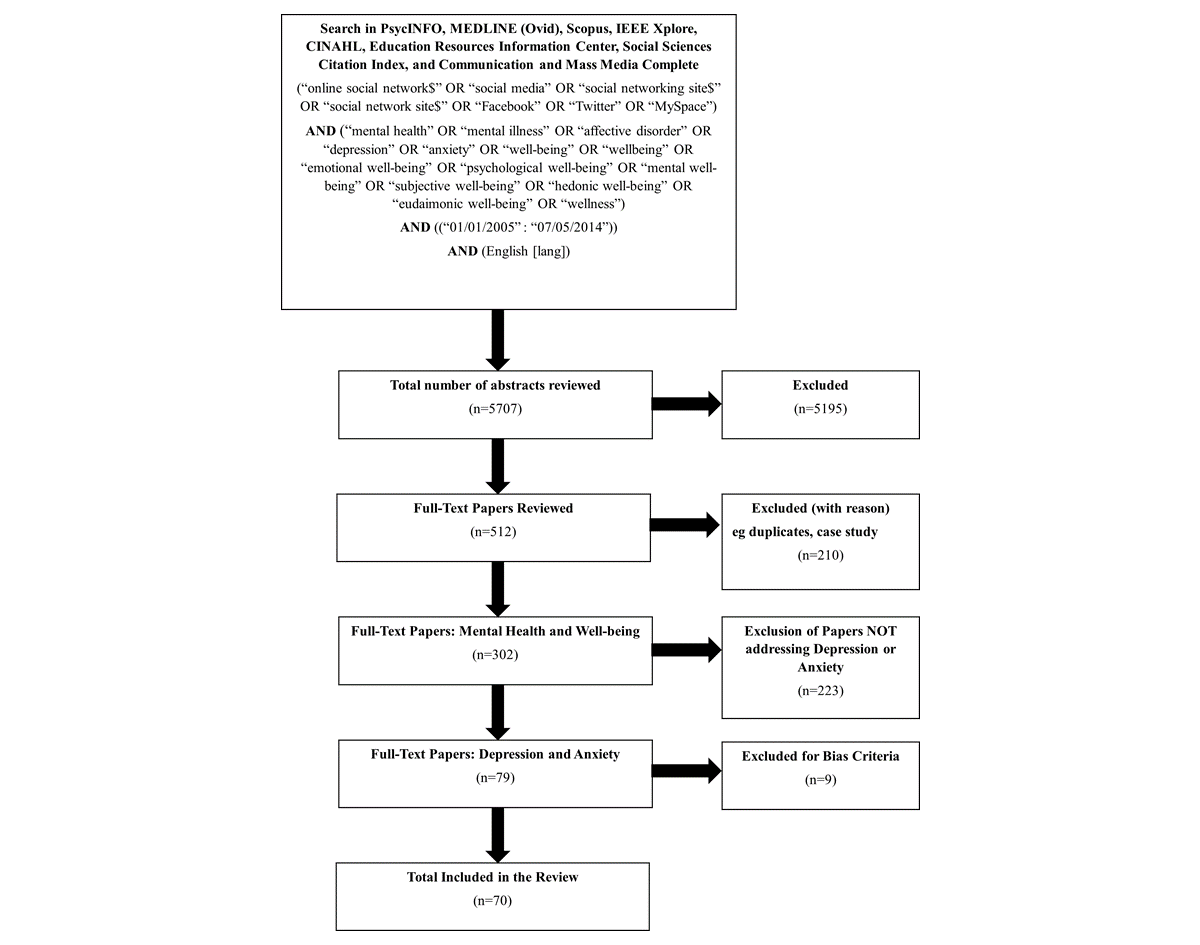
Overview of search strategy and selection process for the systematic review.

Figure 1 summarizes the search strategy and article selection. A multidatabase search identified studies conducted between January 2005 and June 2016. The databases included were PsycINFO, MEDLINE (Ovid), Scopus, IEEE Xplore, CINAHL (Cumulative Index to Nursing and Allied Health Literature), Education Resources Information Center, Social Sciences Citation Index, and Communication and Mass Media Complete. The inclusion of conference papers accessed through IEEE Xplore was intended to capture the research within the computer sciences and engineering fields that may have been relevant to the psychological literature.

Search terms were selected in order to comprehensively capture the various ways mental health, mental illness, subjective well-being, and SNSs have been defined and explored in the existing literature.

SNSs were defined as conceptualized by Ellison and Boyd [1] as sites that are a Web-based communication platform with 3 distinct characteristics: (1) user profiles are unique and created through user-provided content and content provided by other users, (2) the network connections between individuals are visible and can be navigated through by other users, and (3) individuals can broadcast content and consume and interact with content contributed by others in a continuous stream of information. Prototypical examples of SNSs include Facebook, Twitter, Myspace, and Instagram.

For mental health, search terms specifically focused on depression and anxiety, as well as overall well-being (eg, subjective well-being, psychological well-being, wellness; see Figure 1 for full list of search terms).

### Inclusion and Exclusion Criteria

Studies were included if they had a primary focus on SNS use as a behavior. As such, studies that referred to SNSs as a recruitment method only or used SNSs as a means for intervention delivery were excluded.

Articles were included if they provided results addressing anxiety or depression directly and were excluded if they were only referred to in the context of general psychological distress (or similar). As the primary focus of the review was on depression and anxiety, not the broader well-being construct, articles addressing well-being were only included if they also included specific reference to anxiety or depression.

The search was limited to articles published after 2005 to capture research on the prototypical examples of SNSs that include the basic features of modern networks. Studies that had a primary focus on the Internet, chat rooms, or online support forums were also excluded; although they may contain some of the features of SNSs, differences in the function they perform for users may exist [19].

Additionally, articles were restricted to English language, peer-reviewed journal or conference proceedings, and quantitative or mixed methodologies. Gray literature, commentary and editorial, qualitative research, literature reviews, and descriptive case studies were excluded.

### Data Extraction and Data Synthesis

Two raters (the first author and a trained research assistant) reviewed all abstracts returned from the literature search and selected abstracts for full-text reading based on the inclusion and exclusion criteria. All articles that included measurement of depression, anxiety, or well-being were retained. The selected full-text articles were downloaded and reviewed by the first and third authors.

To provide some preliminary evaluation of the strength of the research, three risk of bias indicators were adapted from the Cochrane bias tool (*Cochrane Handbook for Systematic Reviews of Interventions* [43]), which classifies methodology that may limit replicability or generalizability. Studies were rated to indicate whether the study (1) included psychometrically reliable and valid measures, (2) used an external measurement criterion for mental health, and (3) provided description of the sample demographics including some SNS activity statistics (eg, number of friends and/or use frequency). These were rated by the first and third authors from “0=No bias,” “1=Unclear risk of bias,” and “2=High risk of bias” and were summed to create a final score between 0 and 6. A linear weighted kappa statistic for interrater reliability (.78, SE=.06) indicated that there was very good agreement in applying the bias criteria. Consensus was reached on all ratings. Articles with a rating of 3 or above were excluded [44-52], resulting in the final set of 70 studies, as presented in Multimedia Appendix 1.

From each article, the year of study, population of interest, type of SNS, and variables used (anxiety, depression, well-being) were noted, along with whether or not any formal mediators or moderators of these relationships were indicated. Information was then qualitatively synthesized to identify common themes.

## Results

### Description of Studies

**Figure 2:**
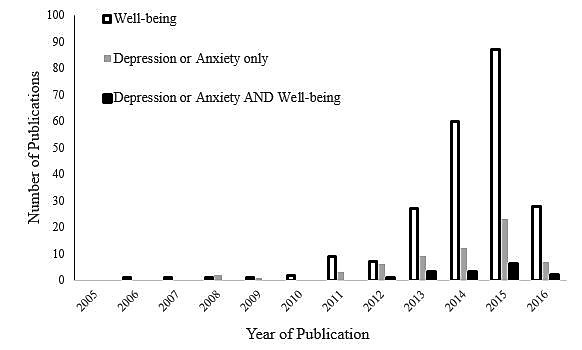
Publication frequency of research into well-being, depression or anxiety only, and depression or anxiety with well-being from 2005 to June 2016, based on the initial 302 full text articles reviewed, which included quantitative findings. Case studies, editorials, literature reviews, and gray literature were excluded.

Figure 2 indicates the number of articles addressing SNSs, depression and anxiety, and well-being from 2005 through 2016, based on the 302 full-text articles initially reviewed. There were considerably more articles addressing well-being alone than articles only addressing depression and anxiety. Only 15 articles included both positive and negative aspects of mental health. This review includes the 70 articles that include depression or anxiety only or depression or anxiety and well-being.

A total of 22 studies addressed potential moderators or mediators in SNSs’ relationship with depression or anxiety (see Multimedia Appendix 1). Most articles obtained a bias rating of 0 to 1. Ratings of 1 or above were primarily due to the limited focus on reporting SNS activity statistics, such as the number of friends or average frequency of use, which help characterize the average SNS user in each sample. Facebook was the most commonly explored SNS followed by the measurement of SNS use as a general category (ie, no specific platform explored). The majority of studies examined young adults (late teens or early 20s).

### Depression, Anxiety, and Social Networking Sites: Summary of Findings

Across the 70 articles, several general themes were apparent: frequency of use, size and structure of the SNS, language features and observable SNS activities, self-disclosure and expression, quality of interactions, social support, social connectivity, social comparison, addictive and problematic behaviors, and physiological associations. Findings are summarized in Multimedia Appendix 2 and are described below, with particular attention to moderators and possible mechanisms involved in the associations. As some articles were relevant to multiple themes, these articles appear in multiple sections. Studies that included well-being are also highlighted.

#### Frequency of Social Networking Site Use

Overall, total frequency or time spent on SNSs had mixed associations with depression and anxiety. Of the 30 studies examining these variables (see Multimedia Appendix 2) [53-81], 8 studies found a direct positive association with depression and 16 found a nonsignificant association. For anxiety (and social anxiety), 3 studies found direct positive associations and 7 found nonsignificant associations. With the exception of 1 study showing a significant negative association between Facebook-specific social anxiety and the frequency of SNS use [80], no studies supported an association between the frequent use of SNSs and a lower level of anxiety or depressive symptoms.

Several moderators appeared. In one study, the number of strangers followed moderated frequent Instagram use and greater depressive symptoms, where a significant relationship only occurred for those with high proportions of strangers in their social networks [68]. Similarly, time spent on Facebook was only a predictor of depression and anxiety for those individuals who have higher motives to use the site for social connection [73].

Associations may be affected by the study design. Studies utilizing an experience sampling method (ESM) to collect SNS use frequency over 1 to 2 weeks found no significant associations between SNS use frequency and depressive symptoms over time [61,63,77]. Indeed, across 2 studies, while Steers et al [77] found a positive association between the time spent on Facebook and depression when using a retrospective survey, this effect was nonsignificant when participants completed daily ESM diaries. In addition, 2 studies [54,56] conducted a 3-week follow-up and demonstrated no change in depressive or anxiety symptoms over time as a function of SNS use frequency.

Tendencies toward depressive rumination and corumination did not moderate associations, suggesting that the frequency of SNS use may not be a significant risk factor for depression even across varying cognitive styles [54]. Kross et al [63] additionally included depression as a moderator of the relationship between the frequency of daily SNS use and affective well-being (ratings of negative affect) and cognitive well-being (life satisfaction). Although more frequent SNS use was associated with more negative affect and lower life satisfaction across a 2-week period, depression did not moderate these associations.

A number of studies have made a more nuanced consideration of SNS use frequency by looking at the different functions of use of SNSs [54,56,69,74-76,78]. Table 1 presents how these broad functions have been defined in the literature and presents some example behaviors. It also provides the Cronbach alphas that have been reported for the measures of each function. The table shows a distinction between passive and active use (broad-level functions). Active use may further be divided into content production and interactive communication functions. The table also shows where behaviors may be enacted in public (entire SNS friend network audience) or in private (dyads or small selected audience).

**Table 1:** Broad functions of social networking site use and example behaviors.

	Passive use (alpha=.77-.88)^a^ [75,78]	Active social use (alpha=.83-.86)^a^ [69,76]	
		Content production (public) (alpha=.52)^a^ [75]	Interactive communication (alpha=.80)^a^ [75]
Example behaviors	Checking or reading friends’ profiles or posts Browsing the newsfeed	Status updates Updating profile pictures Image management (maintaining profile information)	Chatting in messages (private) Posting on friends’ walls (public) Posting comments on statuses (public)

^a^Cronbach alphas indicating the internal consistency of measures defining functions of social networking site use as defined in the reviewed literature.

In general, passive uses of SNSs was not directly related to depression and anxiety, but there may be differential behavioral patterns for individuals high in depression or social anxiety [75,78]. Higher levels of social anxiety were significantly related to passive uses of Facebook but not to content production uses of Facebook [75]. Brooding, or anxious rumination, emerged as a mediator of the relationship between passive Facebook use and social anxiety and may be a cognitive risk factor for increasing social anxiety symptoms where passive Facebook use is frequent. Tandoc et al [78] found that Facebook envy mediated frequent passive Facebook use and depression, where lower levels of Facebook envy resulted in a direct effect of passive Facebook use reducing depressive symptoms and higher levels of envy led to greater depressive symptoms.

Active uses of SNSs demonstrate a more complex relationship. Shaw et al [75] found that depressive symptoms positively correlated with more frequent content production and interactive communications. McCord et al [69] showed that the frequency of social Facebook use did not predict social anxiety in the entire sample but was positively correlated with anxiety for a high anxiety group only.

Simoncic et al [76] suggested that personality and gender moderate the association of frequent active uses of Facebook (content production and interactive communication) and depression and may be protective. The study found a three-way interaction between gender, Facebook active uses, and neuroticism, such that lower depressive symptoms occurred in females who were high in neuroticism and actively used Facebook.

#### Size and Structure of Social Network on Social Networking Sites

The size of the SNS friendship network and its association with depression and anxiety has similarly yielded mixed findings. Fernandez et al [57] and Weidmann and Levinson [82] found significant negative relationships between social anxiety and the number of friends, and Park et al [83], Park et al [84], Rae and Lonborg [73], and Rosen et al [74] found this same relationship direction when examining depression. Rae and Lonborg [73] found that a greater number of friends on Facebook was associated with higher general positive affect and life satisfaction, when use of the site was motivated by maintaining friendships. The remaining studies demonstrated no significant relationship between the number of SNS friends, depression, or anxiety [53,57,64,67,71,73,78,79,85,86].

Specific friend categories have also been examined. Tsai et al [87] found that users accepting the friend request of an ex-partner tend to have higher levels of trait anxiety and depression severity than those who reject the request. Mota-Pereira [88] demonstrated that for individuals with treatment-resistant major depressive disorder (MDD) also currently taking antidepressants, the use of Facebook over a 3-month period significantly reduced depressive symptoms, compared with a no-Facebook control, and the addition of a “psychiatrist as a friend” showed significantly faster improvement in depressive symptoms. Such findings suggest a broad beneficial impact of SNS use when treatment is augmented by friends from a user’s network.

The structure of the network itself may make a difference. For instance, Homan et al [89] revealed significant differences in the network structures of individuals with depression and those without on an LGBTQ (lesbian, gay, bisexual, transgender, and queer) support SNS, TrevorSpace. Individuals without depression had significantly more integrated friendship networks on the SNS compared with depressed individuals, with their friends being more likely to know each other and also having a higher proportion of friends who do not know each other. For the depressed group this could indicate they have less diverse social networks. Peer-selected groups have the potential to offer social support to depressed individuals, whereas groups over which the user had less control may contribute further exposure to psychological distress [90].

#### Language Features and Observable Social Networking Site Activity

A number of articles have examined the language features in SNS posts, with the potential for identifying individuals with depression. SNS users with depression differ from users without depression in that they express negative affect more frequently, use more personal pronouns, and generally have lower frequencies of interaction with others in their SNS network [91,92]. Park et al [93] have shown that individuals with a diagnosis of MDD more frequently post negative sentiment than those who are not depressed, and Moreno and colleagues [85,94] demonstrated that depression could be identified in the language used in the Facebook posts of college students based on the *Diagnostic and Statistical Manual of Mental Disorders* (Fourth Edition) criteria for MDD.

Settani and Marengo [95] directly examined the expressed emotion in participant status updates and generated an automated word count from the emotion dictionaries of the Italian version of *Linguistic Inquiry and Word Count* (*LIWC*), which was also supplemented with emoticons. Providing face validity, the frequency of word use from the negative emotion and sadness *LIWC* subscales positively correlated with depression, while the anger subscale positively correlated with anxiety. Positive emotion was unrelated to depression or anxiety scores. Interestingly, only the relationship between the sadness subscale and anxiety remained statistically significant when examining individuals older than 25 years.

In addition to language features, the time of posting, relative volume of posts, and reciprocity (likes and comments, tweets and retweets) may also aid in describing individuals with and without depression, with depression correlating with more night activity and less volume and reciprocity than nondepressed peers [84,91,96]. Over multiple weeks, there may also be subtle variation across time [96]. Park et al [84] provided evidence indicating that, for individuals experiencing acute depression (or a relative increase in their symptom severity), there is an increase in their posting frequency over a 6-month period. This is consistent with Shaw and colleagues’ [75] findings indicating those with higher depressive symptoms engage in content production features on Facebook frequently.

The number of identity items on SNS users’ profile page have also been associated with both depression and social anxiety scores [57,82,97]. For example, listing a “Single” relationship status relates to higher levels of social anxiety [82]. This related to the quantity of information provided in specific areas of a user’s profile information (eg, TV, Books, Quotes, Music; [57]). Although some of the specific findings are mixed [57,82,98], studies generally suggest that social anxiety may be visible on SNSs through compensatory behaviors (increases in information disclosure) or through relative inactivity or social withdrawal [57,82].

#### Social Networking Sites for Self-Disclosure and Expression

At a broad level, it has been suggested that users of Facebook have lower levels of social anxiety than nonusers, suggesting that there might be a selection effect, such that SNS activities are unattractive to individuals high in social anxiety [99]. However, this depends on the social media platform. Baker and Moore [100] showed that, for new Myspace users, those who intended to use the site for blogging had higher mean depression and anxiety ratings than those who did not intend to blog. These individuals were also more likely than nonbloggers to feel dissatisfaction with their social networks and had a greater likelihood to use self-blame and venting coping strategies. Average levels of depression and anxiety among the bloggers were maintained across a 2-month period, although there was a trend in some symptoms being reduced and a significant increase in feelings of social integration and satisfaction with online and offline friendships [101]. Similarly, große Deters and Mehl [102] found that depressive symptoms remained stable through an intervention, although loneliness decreased via feelings of social connectedness.

Social anxiety is associated with an increased preference for SNS-mediated communication [103] and relates to differences in the depth of self-disclosure via public (status updates) or private (eg, messages) communication on SNSs. For individuals with higher levels of social anxiety, greater importance is placed on the need for reduced social cues and increased controllability of communication [59,104]. This leads to greater disinhibition and Facebook self-disclosure for private SNS communication only and not for public SNS communication [59]. Green et al [59] suggest that this may be related to the trust, audience size, and privacy differences between private and public communication on SNSs, which may position private SNS communication as more attractive and accessible for individuals high in social anxiety. Similarly, Baker and Jeske [80] suggested that assertiveness on Facebook (the ease with which an individual offers opinion or interacts with others) is lower for individuals high in social anxiety compared with those low in social anxiety.

A potential explanation for the self-disclosure activities of individuals with high social anxiety on SNSs may be related to motivations or perceived pressure to present an idealized self-image or to avoid presenting a negative image on SNSs [86,105,106]. Motivations to avoid presenting a negative self-image have been found to be a greater concern for individuals who had experienced high social anxiety the previous day and does not vary according to levels of perceived social competence [105]. Similarly, frequent impression management (including updating profile information) on SNSs is positively related with depression [74].

Frequently expressing positive or negative affect (emotional valence) in SNS status updates has also been shown to relate to depression and may be mediated by rumination [67]. In contrast, positive and negative expression appears to be unrelated to social anxiety [98]. Positive and negative self-disclosures may, instead, impact the quantity of social reciprocity an individual with social anxiety receives [98]. For example, when individuals higher in social anxiety post positive status updates, this generates more pronounced increase in social feedback (likes) than when positive posts are made by those low in social anxiety or when posts have low positive content [98].

#### Quality of Interactions

Considerable evidence suggests a link between the quality of interactions on SNSs and mental health. Studies have operationalized SNS interaction quality as either the perceived (when self-rated) or observed (when coded by experimenters) valence of interactions between friends and the user on SNSs. Items often refer to a global estimate of “How positive [or negative] are your interactions with people on Facebook” [54] or, where coded, the frequency of positive or negative sentiment expressed in comments on posts [103]. This differs from the frequency of social or interactive communication on SNSs, discussed above, which refers to the estimated frequency or total time spent engaging in these activities.

Depression is generally associated with fewer positive interactions and more negative interactions on SNSs [54,56,103,107,108]. Social and global anxiety similarly relate to the perception of negative quality interactions on SNSs [56,107]. Depressed individuals may use SNSs in a more problematic manner than do anxious individuals [56], thus creating negative interactions. For instance, symptoms recorded at the age of 13 years significantly predicted a reduced likelihood of receiving comments that contained deviancy talk from SNS peers at the age of 20 years; however, symptoms at the age of 20 years predicted a greater instance of verbally abusive comments from peers [103]. The findings of Frison et al [81] also suggest that depressive symptoms are a risk factor for peer victimization on Facebook. Moberg and Anestis [108] have additionally shown that, when controlling for the influence of depressive symptoms on perceived negative interactions on SNSs, greater ratings of negative interactions predict feelings of thwarted belongingness (disconnection), a potential risk factor for suicidal desire.

Depressive rumination and corumination may moderate associations between the perception of SNS interaction quality and depression. In 2 studies, Davila et al [54] showed that those with higher levels of depressive rumination exhibited a stronger relationship between the frequency of perceived negative interactions on SNSs and greater depressive symptoms. Although corumination (ie, “excessive discussion of problems within friendships”; [54] p73) did not emerge as a significant moderator, it did yield a number of relationships with other variables, notably, feeling down or depressed after interactions on SNSs and a greater frequency of SNS use. The quality of use also relates to intentions for continued SNS use. Belief that online communities are dangerous, including concerns about privacy and the potential to encounter hostile or negative interactions, has been shown to be a potential antecedent of online and general social anxiety and their link to reduced continuance intention of using Facebook for social communication [109].

Associations may depend in part on the methodologies used. When researchers have directly observed and coded the language of comments made to an SNS user by their friends, it has been shown that a greater level of social anxiety at age 20 years was a significant predictor of more positive supportive comments from SNS friends and fewer negative peer interactions [103]. This is in contrast with the research utilizing self-report survey methods that show more frequent reporting of negative interactions for those with high levels of depression and anxiety symptoms [54,56,107]. This discrepancy suggests there may be a role for perceptual bias in a participant’s interpretation of the quality of interactions to which they are exposed on SNSs. In this light, individuals with higher levels of depression and anxiety may be more inclined to interpret or perceive SNS interaction as more negative regardless of the communication content exchanged between users. The potential for such a perceptual bias in interpreting SNS interactions has also been suggested in reference to social support perceptions and is further discussed below (see Park et al [93]).

#### Social Support

Social support plays a mixed and varied role within the SNS environment. Studies suggest that individuals with higher depressive symptoms perceive their SNS friend networks as providing them with less social support than they actually receive [93] and that SNS social support seeking may exacerbate depressed mood for some individuals [110]. Perception of support appears to be more important than actual support. Across 2 studies, Park et al [93] showed that in the general population greater depressive symptoms were associated with more *actual* social support on status updates that contained negative emotion. In contrast, *perceived* support was negatively associated with depression, and higher depressive symptoms were associated with a greater discrepancy between actual and perceived social support. Frison and Eggermont [110] similarly found that depressed mood increased in adolescents when social support was sought on Facebook but perceived to not occur. Other research has also demonstrated the protective role of perceived social support in ameliorating the impact of SNS peer victimization on depression [81].

For anxiety, social support provided on SNSs may play a protective role. Indian and Grieve [111] found that perceptions of Facebook social support were only predictive of subjective well-being for individuals with high levels of social anxiety and not for those reporting low levels of social anxiety. Furthermore, in the high social anxiety group, perceived Facebook social support was the only significant predictor of subjective well-being, suggesting that SNS social support may provide unique benefits to individuals with high levels of social anxiety.

The nature of seeking social support on SNSs may differ from traditional face-to-face approaches [110,112]. Some evidence suggests that emotional support provided by Facebook can increase depressive symptoms and decrease quality of life [112]. It may depend in part on the characteristics of the user. For example, SNS users’ perceived communication competence—an overall evaluation of communication skills and behaviors—plays a role in determining the level of satisfaction they feel is generated from their SNS social support. Wright et al [79] demonstrated that better perceived communication competence predicted higher ratings of both face-to-face social support and Facebook social support satisfaction, which in turn were significantly negatively related to depression.

#### Social Connectedness

Facebook social connectedness encompasses subjective feelings of belonging and closeness to an individual’s social network [113]. Grieve et al [113] demonstrated that higher levels of Facebook social connectedness were related to lower levels of depression and anxiety and higher levels of subjective well-being (life satisfaction). Feelings of social connectedness may mediate the impact an increase in posting behavior has on decreasing loneliness [102].

#### Social Comparison

Social comparison on SNSs, where individuals compare themselves as having more positive (downward comparison) or negative (upward comparison) qualities than others, is a significant risk factor for depression and anxiety [68,77,114,115]. Several studies found that Facebook envy, a hostile evaluation of others from their social information on SNSs, is associated with higher ratings of depressive symptoms [78,116]. Lee [114] found that depression and anxiety were positively related to the frequency of social comparison on Facebook. Feinstein et al [115] extended these findings by revealing rumination as a mediator in the relationship between negative (upward) social comparison on Facebook and depressive symptoms. This relationship changed over time; at a 3-week follow up, more frequent negative social comparison on Facebook was associated with increases in rumination and a subsequent increase of depressive symptoms.

Appel et al [116] examined how depression may influence an SNS user’s interpretation of the profile information of other users. Individuals with depression were more likely to rate themselves as being unhappier (or inferior) in comparison with profiles of any type (attractive or unattractive) than those without depression. Individuals with depression also experienced greater envy than those without depression in response to viewing the unattractive profile, with this difference being greater after viewing the attractive profile.

Social comparison of any direction (upward, nondirectional, or downward) may also indirectly mediate the association between the time spent on Facebook and depression. Across 2 studies, as individuals spend more time on Facebook they engage in more frequent negative (upward) and nondirectional social comparison and less positive (downward) social comparison, which in turn relates to more depressive symptoms [77].

Envy potentially plays a destructive role in passive Facebook use (eg, viewing or browsing profiles; see Table 1). Where Facebook envy is high, greater frequency of passive Facebook use is associated with greater depressive symptoms, and where Facebook envy is low (or not present), passive Facebook use is associated with reduced depressive symptoms [78]. Indeed, research into Instagram (a photo-sharing SNS) [68] has shown that more positive (downward) social comparisons are associated with decreased depressive symptoms. Social network composition, additionally, may moderate the relationship between frequent Instagram use and increases in depressive symptoms via social comparison [68].

#### Addictive or Problematic Social Networking Site Use

“SNS addiction” and “problematic SNS use” are linked with depression and anxiety [58,60,62,65,104,106,117-121], although associations most likely are bidirectional in nature. It has been suggested that such maladaptive SNS use is only present for a small subset of users [62,106], although one study suggested that 41.9% of adolescents had a Facebook addiction [119]. While depression and social anxiety explain much of the variance in problematic SNS use or SNS addiction, other variables (younger age, male, and more frequent SNS or general Internet use) have also emerged as significant predictors [58,62,118]. Through cluster analysis, Moreau et al [120] showed that problematic Facebook use is most prevalent in individuals high in borderline personality traits and depressive and social anxiety symptoms compared with groups low in those symptoms or high in sensation seeking (but low in psychopathology). Their findings may indicate considerable comorbidity between psychopathological symptoms and SNS addiction.

Wegmann et al [121] suggested that depressive symptoms and social anxiety have both a significant direct relationship with SNS-specific addiction and a partially mediated pathway to SNS-specific addiction via 2 cognitive styles: self-regulation and Internet use expectancies. In these pathways, higher levels of depression and anxiety are related to lower levels of self-regulation, which are in turn related to higher SNS-specific addiction scores. Internet use expectancies, the perception that the Internet can aid in increasing pleasure and decreasing negativity, were greater for those with higher depression or anxiety symptoms, which again lead to greater vulnerability for SNS-specific addiction. They suggest that depression and social anxiety may predispose SNS users to these cognitive styles.

In contrast, Andreassen et al [117] found that while social anxiety was positively related to addictive SNS use, depression was negatively related to addictive SNS use. This was interpreted as reflecting social withdrawal characteristics of depression and CMC’s social compensation for individuals with social anxiety [117]. Indeed, addiction and the compensatory uses of SNSs have been demonstrated to be related to higher levels of social anxiety [106]. Some evidence suggests that the addictive use of SNSs arises from the need to compensate for the social functions affected by social anxiety symptoms. Casale and Fioravanti [104], for example, show that addressing unmet face-to-face social needs, such as the need to belong, to be perceived as socially competent, and to be assertive in communication, may drive problematic SNS use. However, associations may depend on gender. For males and females, a direct association between social anxiety and problematic SNS use has been demonstrated; however, a significant mediator (motivations for competent self-presentation) in this relationship only emerged for males [104]. Lee-Won et al [65] suggested that when the need for social reassurance (ie, motivations to seek social interactions and feelings of belonging) is high or moderate, the relationship between social anxiety and problematic SNS use is strengthened. Thus, social anxiety may only be a risk factor for problematic use of SNSs where the need for social connection is also high.

#### Physiology and Facebook

Finally, one study examined the impact of Facebook or face-to-face exposure as a primer for physiological arousal [122]. Arousal was greater for individuals when observing someone face-to-face after browsing their Facebook profile than for individuals exposed to a face-to-face encounter followed by the Facebook condition. Social anxiety was a significant moderator, with a more pronounced increase in arousal for those high in social anxiety, particularly in the Facebook than face-to-face exposure. The authors suggested that for the high social anxiety group, the initial exposure to Facebook may prime social comparison and self-presentation concerns for the subsequent face-to-face meeting. However, as emotional valence was not measured, it is unclear if the arousal experienced by participants was perceived as a positive or negative event.

## Discussion

### Principal Findings

This systematic review examined associations between SNS use and anxiety and depression. Across 70 studies reviewed, a number of positive and negative correlates have been suggested, as well as moderators and mechanisms of these associations. On the basis of this review, it is likely that there are differing engagement and interactional styles on SNSs for users high in social anxiety and depression. These may be driven or defined by both symptoms and motives to compensate for needs that are not met face-to-face. Negative interactions, frequent social comparison, and SNS addiction or problematic use are related to higher levels of depression and anxiety. Furthermore, cognitive response styles such as rumination or brooding may exacerbate the negative interactions between SNS use, depression, or anxiety for some individuals.

While these potential risks exist for mental health, it is also clear that SNSs can provide considerable benefits to their users. Positive quality interactions, social support, and social connectedness most consistently related to lower levels of depression and anxiety. Social support and connectedness derived from SNS use may be uniquely beneficial to individuals with social anxiety who are unable to access these resources face-to-face. However, especially for those with depression, some evidence suggests that there is a discrepancy between the perceptions of interaction quality and social support and the actual content of their SNS communications, which may attenuate the potential positive impacts of SNS use.

Across a number of studies, observable SNS features such as language use and expressions of identity on user profiles have been demonstrated to provide insight into the depression and anxiety status of the SNS user. With continuing research these characteristics may be a useful tool for monitoring mental health. The content and quality of interactions on SNSs may provide the clearest candidates for monitoring depression and anxiety and may be potential intervention targets for improving mental health and well-being through engaging with SNSs.

### Social Aspects of Social Networking Sites

Across studies, social aspects, including feelings of social support, social connectedness, and positive interaction quality, emerged as protective factors for SNS users. The SNS network structure itself may play an important role in supporting mental health, in that some platforms may better provide social resources to individuals with depression. Indeed, more integrated social networks on SNSs were associated with lower levels of depression [89]. Studies suggest that social support and social connectedness derived from SNSs are constructs distinct from general social support or connectedness [111,113]. SNSs may therefore be contributing additional benefit to their users by creating another domain in which individuals can access, or have greater perceived access to, social support, especially with individuals for whom face-to-face interaction is difficult [123-125]. The broad and visibly articulated social context on SNSs may contribute to the feeling of social connectedness derived from SNSs and its association with better mental health outcomes [126]. As such, SNSs may provide an environment where those already high in social skills and resources are benefiting from their cumulative sources of social support (“rich-get-richer”; see [28]) as well as augmenting social support access for those who have difficulties engaging face-to-face [111,123-125].

Consistent with offline research, the perception of social support appears to be more important than actual support [126-128]. Findings demonstrated that perceived social support was greater in those with lower depression scores and that perceived communication competence may contribute to this relationship [79,93]. Greater perceived positive interaction quality and greater reciprocity in interactions are also indicative of lower depression and anxiety. Similarly, Valkenburg et al [32] demonstrated higher levels of life satisfaction and self-esteem for those who frequently reported positive peer experiences on SNSs. However, aspects of the individual that drive depressive feelings and social anxiety, greater use of negative language, and cognitive aspects such as social comparison and rumination, can prevent the user from perceiving support that is actually there [93], further contributing to depressive or anxious symptoms.

### Emotional Aspects of Social Networking Sites

The valence of posts on SNSs may both reflect and impact depression and anxiety. Individuals scoring higher on depression scales in the reviewed studies generally expressed more negative affect on SNSs and were more likely to perceive negative interactions. The way individuals interpret emotional and social content on SNSs may place depression as antecedent to maladaptive SNS use, which may, in turn, maintain depressive symptoms. For individuals who are already depressed, ambiguous interactions are often interpreted as negative [13,129], which may attenuate the potential benefits available through SNS use.

Evidence suggests that frequent positive expressions are associated with better mental health, and frequent negative expressions are associated with depression and poorer life satisfaction [67,91,96]. While therapeutic writing can provide some benefits in reducing distress and improving well-being [30,31], online writing may serve a different function, with Web-based expressions reflecting the lived experience of the individual (eg, [91,130-132]), rather than providing a therapeutic outlet. Indeed, relative increases in posting frequency were shown to be associated with greater depressive symptoms [84]. For others, the presence of social anxiety may hinder the use of posting functions for emotional disclosure on SNSs [59], which may decrease access to potential social interaction [98]. As emotional content can be effectively communicated on the Web [133], SNSs represent another space in which positive and negative interactions can be enacted and may provide key behavioral insights into the mental health and well-being of a SNS user. Alternatively, increases in self-expression on SNSs may be more beneficial to well-being domains (such as connectedness, social support, and life satisfaction) but may not have an impact on depression or anxiety. A direct comparison of these relationships has not been conducted, and might be an area to investigate in the future.

### Cognitive Aspects as Mechanisms and Moderators

The prominent risk factors for depression and anxiety that emerged from this review included frequent SNS social comparison, negative perceived interaction quality, addictive or problematic SNS use, and rumination (or brooding). These factors represent cognitive and interactional styles that have well-established associations with depression and anxiety but may be enhanced by the enduring nature of social content on SNSs. Although the total frequency of SNS use does not appear to be directly related to either depression or anxiety, there are different moderating and mediating factors [68,73,77,78] and patterns in the functions of SNS use by individuals with higher depression or anxiety that may contribute to or exacerbate symptoms [69,74-76,78].

One of the risk factors for depression and an individual’s interaction with SNSs was rumination. Greater rumination is frequently associated with higher ratings of depression and also impacts well-being by maintaining a focus on negative affect [134,135]. Rumination is a likely mechanism for the relationship between negative interactions with SNSs and depression based on its role in SNS negative emotional expression [67] and social comparison [115]. There is considerable potential for SNSs to amplify and assist ruminative processes by exposing SNS users to a constant stream of rich social information that can be selectively reflected on as permanent content on a user’s profile [54,115].

Similar to depression, the cognitive risk factors for social anxiety include social comparison (via brooding) and the perception of frequent negative interactions. However, the pathway to and importance of these risk factors may differ from depression. In contrast to those with depression, those high in social anxiety mainly use SNSs for passive browsing and private communication, not for content production [75]. The passive uses of SNSs may place individuals at greater risk of more frequent social comparison, which may have negative mental health effects [114]. This differs from the relative benefit of content production on SNSs for an individual with social anxiety, as posts are often rated as being more appreciated by friends in the network [98], which may have a flow-on effect to the perception of SNS-derived social support [111] and may even reflect more positive interactions with peers [103].

The reduced social cues on SNSs may be attractive to individuals with social anxiety, as has previously been suggested in the general Internet literature [124]. However, the need to compensate for a lack of belonging and social reassurance in face-to-face interactions, in conjunction with lower self-regulation, may drive problematic SNS use for individuals with social anxiety [65,104,106,117]. Similarly, these motives may also contribute to individuals with social anxiety generating more content on their profile pages than others [57], and for those highest in social anxiety it may contribute to a higher frequency of SNS use [69]. On the whole, there appear to be a number of well-being benefits to using SNSs for individuals high in social anxiety that cannot be gained in face-to-face interactions; however, the pattern of SNS use may negatively affect other domains.

### Mixed Results and Nonpredictors

The frequency of SNS use as a whole suggested no clear association with depression and anxiety. Longitudinal research suggests that depression and anxiety remain stable in the context of how frequently a user engages with SNSs [54,56,61,63,77] and the function of use holds clearer associations with depression and anxiety [75]. This is consistent with the literature examining general Internet use where total frequency of use is often not a predictor of depression, particularly when examining the social features of the Internet [28,125]. For example, when examining different functions on the Internet, Morgan and Cotten [29] showed that more hours spent using the Internet for social activities (IM’ing, chat rooms) are associated with decreased levels of depression and that informational uses and gaming are associated with increases in depression.

While total SNS use may not affect psychopathology, it may be related to subjective well-being. This was illustrated in the study by Kross et al [63], in which more frequent SNS use was related to experiencing more negative affect and reducing life satisfaction. As frequent experience of negative affect may contribute to the onset and maintenance of depression, it is likely that a pathway to poorer mental health outcomes exists via the impact SNS use has on the frequency of experiencing positive and negative emotions [54,63,67]. Additionally, other SNS features and cognitive processes (eg, network size, structure, and composition, tendency to ruminate, frequent social comparison) may be more informative in describing the impact frequent SNS use has on mental health.

In contrast with the literature examining social network size and structure offline [12,136], SNS friendship network size, on the whole, was not associated with depression or anxiety. However, some evidence has shown distinct network structure differences between individuals with depression and those without in terms of the interconnection between friends within a network [84]. Individuals with depression or anxiety have previously been shown to have more impoverished social networks, and changes in mental health are often associated with changes in an individual’s social network [12,137]. Impoverished social networks are often a risk factor for depression and anxiety by reducing access to “buffering” social support and increasing feelings of isolation [138-140]. They may also result from poor-quality social interactions, often typical of depression and anxiety [137].

The absence of a clear association between depression or anxiety and the number of friends on SNSs may be explained by one of the major differences between the offline and online social networks; that is, the way friendships are maintained over time. As SNSs do not necessitate direct social interaction to maintain the status of “friendship,” many users may not actively redefine their networks [141]. It is likely that the social pruning and the dissolution of social ties associated with mental illnesses such as depression and anxiety may not be visible on SNSs. Social pruning does occur for many SNS users (eg, 63% of American SNS users endorsed that they had removed friends from the “friends” list; [141]), but how comprehensively this behavior is performed remains unknown. Therefore, change in mental health status for SNS users may not be as accurately detected by a decreased social network size online as it may be when observing offline networks. Other metrics, such as communication output and reciprocity, may be more informative in describing the social network changes associated with depression and anxiety. For instance, De Choudhury et al [91] demonstrated that the volume of tweets and the associated replies were reduced in Twitter users with depression compared with those without.

### Strengths and Limitations

As with any study, there are both strengths and limitations of this review. We included a basic criterion for bias that focused on evaluating the methodology of studies, which considered whether papers included (1) the use of psychometrically reliable and valid measures; (2) an external measurement criterion for mental health; and (3) description of sample demographics that included basic SNS user activity statistics. Only 9 studies were excluded for bias, suggesting that there is relative strength in defining the variables of interest in this field. However, a greater focus on defining the SNS characteristics of the sample is required.

The review attempted to characterize the research in terms of the populations and specific SNSs that have been studied. Studies have focused rather narrowly on the young adult population. While these individuals tend to represent the highest membership category of SNSs, recent estimates have suggested that SNS use is becoming more evenly represented across the life span, with more than 50% of older Internet users (65+ years) now also using SNSs [7]. This is an important consideration for future research as the social connection that may be gained through SNSs may provide more benefit for older users as quality of the interactions, particularly through language use, may vary significantly over the life span [142].

Despite the systematic approach to this review, the identified themes are not exhaustive. Other themes such as the differences between SNS users and nonusers and SNS use motives may have been extracted and more explicitly discussed. The discussion of results was limited to the depression or anxiety context and did not discuss findings outside this scope. Well-being, which clearly is becoming a growing area of interest (Figure 1), was only included if there was also a focus on depression or anxiety. Future studies might extend to other aspects of mental illness and wellness.

Finally, although we identified some moderating characteristics, few studies have considered individual differences such as gender and personality and their interaction with SNS variables. Future studies might give greater attention to how characteristics of users impact the identified factors.

### Implications and Future Directions

The results of this systematic review have revealed considerable support for the importance of examining the content and quality of the interactions a user has with SNSs. As such, the language used in interactions on SNSs could become a target of interest, particularly as it has been shown to be sensitive in identifying individuals with depression [91,92,94,143]. Further research should also focus on the interplay between the network structure components and dynamic interactions observable on SNSs. The SNS friend structure could be instrumental in defining the type and efficiency with which social resources may be accessed on SNSs. Examining network structure in concert with the quality of interactions, characteristics such as perceived social support, and mental health could provide rich explanations for why some people benefit from SNS use and others are placed at risk, echoing the detailed social network research that has occurred offline (eg, [12]).

Only a few studies in this review utilized SNS-derived data to answer their research questions. The majority focused on the use of self-report survey and relied on participant estimates of their SNS behaviors, which may have introduced considerable retrospective bias. This bias was addressed to some extent by including ESMs that more accurately sample a participant’s lived experience [144]. The studies directly observing SNS behaviors indicate that the mental health status of SNS users may be at least partly derived from their patterns of use, language expression, and profile information. These findings provide more weight to the potential of using computational science techniques within psychological research, particularly in characterizing well-being in large community samples [33-35,145,146], as well as predicting personality [147]; see also [148]. In reference to depression and anxiety, SNS data hold huge potential for early identification and time-sensitive monitoring of symptoms [143]. SNS data should be leveraged in future research as a part of ESMs to provide real-time, unobtrusive accounts of social behavior in a natural setting.

### Conclusions

This systematic review examined the recent research on associations between SNSs and depression and anxiety. It examined findings in association with the suggested mediators and moderators and the links made with well-being. With more than 50% of adults using multiple SNSs [7], they permeate many aspects of daily life. For many, SNSs represent a way to socially connect with others. However, for others, SNSs may encourage and perpetuate maladaptive tendencies. SNSs maintain and reflect the complexities of the offline social environment and the risks and benefits it may pose to mental health. SNSs represent a novel, unobtrusive, real-time way to observe and leverage mental health and well-being information in a natural setting, with the ultimate potential to positively influence mental health.

## Appendix

Multimedia Appendix 1 Summary of studies included in the systematic review.

Multimedia Appendix 2 Main results table: associations between depression, anxiety, and social networking site outcomes across the 70 reviewed studies.

## Abbreviations

CMC: computer-mediated communication
ESM: experience sampling method
LIWC: Linguistic Inquiry and Word Count
MDD: major depressive disorder
SNS: social networking site
