# Supplementary material for: Social Networking Sites, Depression, and Anxiety: A Systematic Review
Source: JMIR Ment Health. 2016 Nov 23;3(4):e50. doi: 10.2196/mental.5842 (PMC5143470; doi:10.2196/mental.5842)
Supplement: Multimedia Appendix 1 [file mental_v3i4e50_app1.pdf]

Study	Population	SNS	Sample Size	Bias Rating	Well-being	Mediators/ moderators
Andreassen et al. [117]	General	General	23533	1		
Appel et al. [116]	General	Facebook	89	1	✓	
Baker & Jeske [80]	General	Facebook	184	0	✓	
Baker & Moore [100]	General	MySpace	134	1		
Baker & Moore [101]	General	MySpace	58	1		
Banjanin et al. [53]	Adolescents	Facebook	336	0		
Blachnio et al. [118]	General	Facebook	672	1		
Bodroža & Jovanović [106]	General	Facebook	804	0		
Burke & Ruppel [105]	Young adults	Facebook	152	1		✓
Casale & Fioravanti [104]	Young adults	Facebook	400	1		✓
Davidson & Farquhar [86]	Young adults	Facebook	336	1		
Davila et al. [54]	Young adults	Facebook/MySpace	718	0		✓
De Choudhury et al. [91]	General	Twitter	489	1		
De Choudhury et al. [92]	Other	Facebook	165	0		
große Deters & Mehl [102]	Young adults	Facebook	86	0	✓	✓
große Deters et al. [98]	Young adults	Facebook	362	1		✓
Dumitrache et al. [97]	Adolescents	Facebook	123	2		
Farahani et al. [55]	Young adults	Facebook	265	1		
Feinstein et al. [115]	Young adults	Facebook	268	1		✓
Feinstein et al. [56]	Young adults	Facebook/MySpace	301	0		
Fernandez et al. [57]	Young adults	Facebook	62	0		
Frison & Eggermont [110]	Adolescents	Facebook	910	1	✓	✓
Frison et al. [81]	Adolescents	Facebook	1621	0	✓	✓
Ghosh & Dasgupta [99]	General	Facebook	120	2	✓	
Giota & Kleftaras [58]	Young adults	Facebook	143	1		
Green et al. [59]	General	Facebook	306	0		
Grieve et al. [113]	Young adults	Facebook	618	1	✓	
Hanprathet et al. [119]	Adolescents	Facebook	832	1		
Homan et al. [89]	Young adults	Trevorspace	195	1		
Hong et al. [109]	Young adults	Facebook	230	0		✓

Study	Population	SNS	Sample Size	Bias Rating	Well-being	Mediators/ moderators
Hong et al. [60]	Young adults	Facebook	241	1	✓	✓
Indian & Grieve [111]	General	Facebook	299	0	✓	✓
Jelenchick et al. [61]	Young adults	Facebook	190	0		
Koc & Gulyagci [62]	Young adults	Facebook	447	0		
Kross et al. [63]	General	Facebook	82	0	✓	✓
Labrague [64]	Young adults	Facebook	76	0		
Landoll et al. [107]	Young adults	General	430	1		
Lee [114]	Young adults	Facebook	191	2	✓	
Lee-Won et al. [65]	Young adults	Facebook	243	0		✓
Lin et al. [66]	Adults	General	1787	0		
Locatelli et al. [67]	Young adults	Facebook	251	0	✓	✓
Lup et al. [68]	Young adults	Instagram	117	0		✓
McCloskey et al. [112]	Young adults	Facebook	633	1	✓	
McCord et al. [69]	Young adults	Facebook	216	0		
Moberg & Anestis [108]	Young adults	General	305	0		
Mok et al. [70]	General	General	59	2		
Moreau et al. [120]	Adolescents	Facebook	456	1		
Moreno et al. [85]	Young adults	Facebook	200	2		
Moreno et al. [94]	Young adults	Facebook	307	1		
Morin-Major et al. [71]	Adolescents	Facebook	88	0	✓	
Mota-Pereira [88]	Clinical (depression)	Facebook	60	2		
Pantic et al. [72]	Adolescents	General	160	1		
Park et al. [83]	Young adults	Facebook	55	2		
Park et al. [84]	General	Facebook	212	0		
Park et al. [93]	Young adults	Facebook	103	0		
Rae & Lonborg [73]	Young adults	Facebook	119	0	✓	✓
Raunch et al. [122]	Young adults	Facebook	26	1		
Rosen et al. [74]	General	Facebook	1143	1		
Settani & Marengo [95]	Adults	Facebook	201	0		
Shaw et al. [75]	Young adults	Facebook	75	0		✓

Study	Population	SNS	Sample Size	Bias Rating	Well-being	Mediators/ moderators
Simoncic et al. [76]	Young adults	Facebook	237	0		✓
Steers et al. [77]	Young adults	Facebook	332	0		✓
Szwedo et al. [103]	Young adults	Facebook/MySpace	138	1		
Takahashi et al. [90]	General	Depression specific SNS (unnamed)	105	1		
Tandoc et al. [78]	Young adults	Facebook	736	0		✓
Tsai et al. [87]	General	Facebook	202	0		
Tsugawa et al. [96]	General	Twitter	209	0		
Wegmann et al. [121]	Young adults	General	334	1		✓
Weidmann & Levinson [82]	Young adults	Facebook	77	0		✓
Wright et al. [79]	Young adults	Facebook	361	0		

Note. SNS = social network site. Check indicates that well-being or mediators/ moderators were included. See main article for references.
