# Supplementary material for: Social Networking Sites, Depression, and Anxiety: A Systematic Review
Source: JMIR Ment Health. 2016 Nov 23;3(4):e50. doi: 10.2196/mental.5842 (PMC5143470; doi:10.2196/mental.5842)
Supplement: Multimedia Appendix 2 [file mental_v3i4e50_app2.pdf]

	Study	Mental Health Focus	Key SNS Variables	Direct Associations	Additional Notes and Complex Relationships
**Frequency of SNS use**					
	Banjanin et al. [53]	Depression	Average time on SNS (timeframe unclear)	NS	
	Davila et al. [54]	Depression	Daily average time on SNS	NS	
	Farahani et al. [55]	Depression	Daily average time on SNS	NS	
	Feinstein et al. [56]	Depression	Daily average time on SNS	NS	
	Frison et al. [81]	Depression	Daily average time on SNS	+	
	Giota & Kleftaras [58]	Depression	Daily average time on SNS	NS	
	Jelenchick et al. [61]	Depression	Daily average time on SNS	NS	
	Labrague [64]	Depression	Daily average time on SNS	+	
	Lin et al. [66]	Depression	Daily average time on SNS	+	
	Locatelli et al. [67]	Depression	Daily average time on SNS	NS	
	Lup et al. [68]	Depression	Daily average time on SNS	+	Moderated by the proportion of strangers in a network and was only significant at the highest level. It became NS at the lower levels of strangers followed.

Study	Mental Health Focus	Key SNS Variables	Direct Associations	Additional Notes and Complex Relationships
Pantic et al. [72]	Depression	Daily average time on SNS	+	
Rae & Lonborg [73]	Depression	Daily average time on SNS	NS	Significant interaction-relationship is positive for those higher in motivations to use Facebook for connection purposes
Simoncic et al. [76]	Depression	Daily average time on SNS	NS	
Steers et al. [77]	Depression	Daily average time on SNS	+ (cross-sectional)<br><br>NS (ESM diaries)	Social comparison was a mediator
Tandoc et al. [78]	Depression	Daily average time on SNS	NS	
Hong et al. [60]	Depression	Daily average time on SNS (apps, news feed, chat)	NS	
Davila et al. [54]	Depression	Daily average time on SNS interacting with others	NS	
Rosen et al. [74]	Depression	Frequency of SNS use	NS	
Shaw et al. [75]	Depression	Frequency of SNS use	NS	

Study	Mental Health Focus	Key SNS Variables	Direct Associations	Additional Notes and Complex Relationships
Shaw et al. [75]	Depression	Frequency of SNS Interactive Communication Use	+	
Shaw et al. [75]	Depression	Frequency of SNS Content Production Use	+	
Shaw et al. [75]	Depression	Frequency of Passive SNS use	NS	
Tandoc et al. [78]	Depression	Frequency of Passive SNS use	NS	Significant negative association with depression when mediated by Facebook envy
Rosen et al. [74]	Depression	Frequency of impression/profile management on SNS	+	
Simoncic et al. [76]	Depression	Frequency of Active SNS use	NS	Active uses of Facebook were associated with lower depression in a three-way interaction also including gender and neuroticism (female, high neuroticism).
Davila et al. [54]	Depression	Number of times checking SNS per day	NS	
Lin et al. [66]	Depression	Number of times checking SNS per week	+	

Study	Mental Health Focus	Key SNS Variables	Direct Associations	Additional Notes and Complex Relationships
Koc & Gulyagci [62]	Depression	Weekly average time on SNS	NS	
Mok et al. [70]	Depression	Weekly average time on SNS	*	No inferential statistics are presented
Morin-Major et al. [71	Depression	Weekly average time on SNS	NS	Findings were also NS in reference to the frequency of peer interactions and self-presentation behaviours
Wright et al. [79]	Depression	Weekly average time on SNS	+	
Farahani et al. [55]	Anxiety	Daily average time on SNS	+	
Labrague [64]	Anxiety	Daily average time on SNS	+	
Rae & Lonborg [73]	Anxiety	Daily average time on SNS	NS	Significant interaction-relationship is positive for those higher in motivations to use Facebook for connection purposes
Feinstein et al. [56]	Anxiety	Daily average time on SNS interacting with others	NS	
Koc & Gulyagci [62]	Anxiety	Weekly average time on SNS	NS	
Baker & Jeske [80]	Social Anxiety	Daily average time on SNS	NS	

Study	Mental Health Focus	Key SNS Variables	Direct Associations	Additional Notes and Complex Relationships
Baker & Jeske [80]	Social Anxiety (Facebook-specific)	Daily average time on SNS	-	
Green et al. [59]	Social Anxiety	Daily average time on SNS	NS	
Lee-Won et al. [65]	Social Anxiety	Daily average time on SNS	NS	
Feinstein et al. [56]	Social Anxiety	Daily average time on SNS interacting with others	NS	
Fernandez et al. [57]	Social Anxiety	Frequency of SNS use	NS	
Shaw et al. [75]	Social Anxiety	Frequency of SNS use	+	When included in regression with other functions of SNS use this becomes NS
McCord et al. [69]	Social Anxiety	Frequency of Social SNS use	NS	Significant positive association when moderated by the degree of ‘Anxiety on Facebook’ only for those in the high group.
Shaw et al. [75]	Social Anxiety	Frequency of SNS Interactive Communication	NS	
Shaw et al. [75]	Social Anxiety	Frequency of SNS Content Production	NS	

Study	Mental Health Focus	Key SNS Variables	Direct Associations	Additional Notes and Complex Relationships
Shaw et al. [75]	Social Anxiety	Frequency of Passive SNS Use	+	Remains significant when controlling for depression and anxiety symptoms and was mediated by brooding.<br><br>An alternative mediation model provided support for social anxiety as a mediator of passive Facebook use and brooding.
Burke & Ruppel [105]	Social Anxiety	Weekly average time on SNS	NS	
Frison et al. [81]	*Life Satisfaction*	Daily average time on SNS	-	
Locatelli et al. [67]	*Life Satisfaction*	Daily average time on SNS	NS	
Rae & Lonborg [73]	*Psychological Well-being*	Daily average time on SNS	NS	
Baker & Jeske [80]	*Self-Esteem*	Daily average time on SNS	NS	
Hong et al. [60]	*Self-Esteem*	Daily average time on SNS (apps, news feed, chat)	NS	
Kross et al. [63]	*Life Satisfaction*<br>*Affective well-being*	Frequency of SNS use	-	Depressive symptoms did not moderate these relationships
Morin-Major et al. [71	*Self-Esteem*	Weekly average time on SNS	NS	

	Study	Mental Health Focus	Key SNS Variables	Direct Associations	Additional Notes and Complex Relationships
**Size and Structure of SNS**					
	Banjanin et al. [53]	Depression	Number of Facebook Friends	NS	
	Fernandez et al. [57]	Depression	Number of Facebook Friends	NS	
	Labrague [64]	Depression	Number of Facebook Friends	NS	
	Locatelli et al. [67]	Depression	Number of Facebook Friends	NS	
	Morin-Major et al. [71	Depression	Number of Facebook Friends	NS	
	Park et al. [84]	Depression	Number of Facebook Friends	-	Group Differences: depressed participants had significantly fewer Facebook friends than non-depressed participants
	Park et al. [83]	Depression (BDI)	Number of Facebook Friends	-	NS when depression was measured by the CES-D
	Rae & Lonborg [73]	Depression	Number of Facebook Friends	-	
	Rosen et al. [74]	Depression	Number of Facebook Friends (index)	-	Technology attitudes and anxiety were held constant.
	Tandoc et al. [78]	Depression	Number of Facebook Friends	NS	
	Wright et al. [79]	Depression	Number of Facebook Friends	NS	

Study	Mental Health Focus	Key SNS Variables	Direct Associations	Additional Notes and Complex Relationships
Homan et al. [89]	Depression	Network Structure Characteristics	Differ in high and low depression groups	High depression scores have significantly less integrated online social networks
Takahashi et al. [90]	Depression	Network Structure Characteristics		Described network characteristics and connections across different levels of depression severity. Qualitative analysis is also included
Mota-Pereira [88]	Depression	Facebook use with psychiatrist as a “friend”	-	“Facebook Use” group and “Facebook Use with a psychiatrist as a friend” group, depressive symptoms decreased significantly over a 3-month period compared to a control group.
Tsai et al. [87]	Depression	Accepting friend requests from former partners	+	
Tsai et al. [87]	Anxiety	Accepting friend requests from former partners	+	
Labrague [64]	Anxiety	Number of Facebook Friends	NS	
Rae & Lonborg [73]	Anxiety	Number of Facebook Friends	NS	

	Study	Mental Health Focus	Key SNS Variables	Direct Associations	Additional Notes and Complex Relationships
	Davidson & Farquhar [86]	Anxiety (Facebook-specific)	Number of unique groups on Facebook	NS	
	Davidson & Farquhar [86]	Social Anxiety	Number of unique groups on Facebook	NS	
	Fernandez et al. [57]	Social Anxiety	Number of Facebook Friends	-	
	Weidmann & Levinson [82]	Social Anxiety	Number of Facebook Friends	-	
	Locatelli et al. [67]	*Life Satisfaction*	Number of Facebook Friends	NS	
	Rae & Lonborg [73]	*Life Satisfaction*	Number of Facebook Friends	NS	Significant interaction – relationship is positive for those high in friendship motivations for using Facebook
	Rae & Lonborg [73]	*Positive Affect*	Number of Facebook Friends	+	
	Morin-Major et al. [71	*Self-Esteem*	Number of Facebook Friends	NS	
**Language Features and Observable SNS Activity**					
	De Choudhury et al. [91]	Depression	Twitter Use Data	Predictive language and SNS use features identified	

Study	Mental Health Focus	Key SNS Variables	Direct Associations	Additional Notes and Complex Relationships
Tsugawa et al. [96]	Depression	Twitter Use Data	Predictive language and SNS use features identified	
De Choudhury et al. [92]	Postpartum Depression	Facebook Use Data	Predictive language and SNS use features identified	
Dumitrache et al. [97]	Depression	Identity items on profile	+	
Fernandez et al. [57]	Depression	Identity items on profile	+	Depression was associated with a greater amount of profile information posted
Moreno et al. [85]	Depression	Status update content	Predictive language	Depression symptoms can be observed and coded based on the DSM-IV criteria for MDD
Moreno et al. [94]	Depression	Status update content	+	Depression displays in status updates (based on the DSM-IV criteria for MDD) were associated with PHQ-9 scores

Study	Mental Health Focus	Key SNS Variables	Direct Associations	Additional Notes and Complex Relationships
Park et al. [84]	Depression	Inbound comments	NS	Group differences- fewer inbound comments to depressed participants compared to non-depressed participants
Park et al. [84]	Depression	Inbound likes	NS	Group differences- fewer inbound likes to depressed participants compared to non-depressed participants
Park et al. [84]	Depression	Outbound comments	-	Group differences- fewer outbound comments from depressed participants compared to non-depressed participants
Park et al. [84]	Depression	Wall post rate	+	
Park et al. [93]	Depression	Positive disclosure in status updates	NS	Group differences- fewer between those with MDD and those without. MDD participants disclose more negative and less positive content than non-depressed participants.
Park et al. [93]	Depression	Negative disclosure in status updates	NS	

Study	Mental Health Focus	Key SNS Variables	Direct Associations	Additional Notes and Complex Relationships
Settani & Marengo [95]	Depression	Positive emotion in status updates	NS	
Settani & Marengo [95]	Depression	Negative emotion in status updates	+	
Settani & Marengo [95]	Anxiety	Positive emotion in status updates	NS	
Settani & Marengo [95]	Anxiety	Negative emotion in status updates	+	
Fernandez et al. [57]	Social Anxiety	Identity items on profile	+	Social interaction anxiety was associated with a greater amount of profile information posted
Fernandez et al. [57]	Social Anxiety	Number of posts by friends	NS	
Fernandez et al. [57]	Social Anxiety	Number of status updates	NS	
große Deters et al. [98]	Social Anxiety	Number of status updates	NS	
große Deters et al. [98]	Social Anxiety	Number of likes on status updates	NS	

	Study	Mental Health Focus	Key SNS Variables	Direct Associations	Additional Notes and Complex Relationships
	Weidmann & Levinson [82]	Social Anxiety	Facebook Profile Inactivity	NS	Group Differences- participants listing a single relationship status compared to a relationship with other people and an absence of status updates compared to profiles with status updates had significantly higher scores of social anxiety.
	Weidmann & Levinson [82]	Social Anxiety	Observer ratings of social anxiety from profile content	+	
**SNS for Self-disclosure and expression**					
	Baker & Moore [100]	Depression	Intention to blog	+	Group differences- intending bloggers had significantly higher depression scores than non-bloggers
	Baker & Moore [101]	Depression	Blogging on MySpace	NS	Bloggers (and non-bloggers) had no change in depression scores between T0 and T1 (2-month interval)
	große Deters & Mehl [102]	Depression	Increased status posting	NS	

Study	Mental Health Focus	Key SNS Variables	Direct Associations	Additional Notes and Complex Relationships
Locatelli et al. [67]	Depression	Frequency of negative status updates	+	Rumination indirectly mediated the association between negative posts and depression
Locatelli et al. [67]	Depression	Frequency of positive status updates	NS	Rumination indirectly mediated the association between positive posts and depression (significant negative association)
Baker & Moore [100]	Anxiety	Intention to blog	+	Group differences- intending bloggers had significantly higher anxiety scores than non-bloggers
Baker & Moore [101]	Anxiety	Blogging on MySpace	NS	Bloggers (and non-bloggers) had no change in anxiety scores between T0 and T1 (2-month interval)
Davidson & Farquhar [86]	Anxiety (Facebook-specific)	Self-presentation (role conflict)	+	
Baker & Jeske [80]	Social Anxiety	Assertiveness on SNS	-	Group differences – Higher social anxiety has lower assertiveness scores than low social anxiety

Study	Mental Health Focus	Key SNS Variables	Direct Associations	Additional Notes and Complex Relationships
Casale & Fioravanti [104]	Social Anxiety	Assertiveness on SNS	+	Both males and females have a positive association between social anxiety and the feelings of assertiveness in SNS communication compared to face-to-face settings.
Bodroža & Jovanović [106]	Social Anxiety	Self-presentation	+	
Burke & Ruppel [105]	Social Anxiety	Self-presentation	+	Higher social anxiety was associated with greater concern of presenting a negative self-image on Facebook
Casale & Fioravanti [104]	Social Anxiety	Self-presentation	+	Both males and females have a positive association between social anxiety and the feeling they have more control over SNS self-presentation compared to face-to-face settings.
Davidson & Farquhar [86]	Social Anxiety	Self-presentation (role conflict)	+	

Study	Mental Health Focus	Key SNS Variables	Direct Associations	Additional Notes and Complex Relationships
große Deters et al. [98]	Social Anxiety	Social Reciprocity and Valence of Status Updates	+ (Study 1)<br><br>NS (Study 2)	Positive posts elicit a greater number of ‘likes’, particularly for those high in social anxiety. This was not supported in Study 2
Ghosh & Dasgupta [99]	Social Anxiety	SNS membership	-	Group differences – Facebook users had significantly lower social anxiety scores than non-users. This difference was more pronounced for female non-users compared to males.
Green et al. [59]	Social Anxiety	SNS self-disclosure (public)	NS	
Green et al. [59]	Social Anxiety	SNS self-disclosure (private)	NS	Significant mediated pathway from social anxiety to private Facebook self-disclosure via the characteristics of online communication and disinhibition.
Locatelli et al. [67]	*Life Satisfaction*	Frequency of negative status updates	-	Rumination indirectly mediated the association between negative posts and life satisfaction

	Study	Mental Health Focus	Key SNS Variables	Direct Associations	Additional Notes and Complex Relationships
	Locatelli et al. [67]	*Life Satisfaction*	Frequency of positive status updates	NS	Rumination indirectly mediated the association between positive posts and life satisfaction (significant positive association)
	große Deters & Mehl [102]	*Loneliness*	Increased status posting	+	Loneliness significantly decreased in the experimental group (increased posting) from T1 to T2 (1-week interval) and did not change in the control condition. The decrease in loneliness was mediated by feelings of social connectedness
	Ghosh & Dasgupta [99]	*Self-Esteem*	Facebook membership	+	Group differences – Facebook users had significantly higher self-esteem scores than non-users
	große Deters & Mehl [102]	*Subjective Happiness*	Increased status posting	NS	
**Quality of Interactions**					
	Davila et al. [54]	Depression	Perceived frequency of negative interactions	+	Greater perceived frequency of negative interactions (T1) predicted increases in depressive symptoms (T2)

Study	Mental Health Focus	Key SNS Variables	Direct Associations	Additional Notes and Complex Relationships
Davila et al. [54]	Depression	Perceived frequency of positive interactions	NS (Study 1)<br>- (Study 2)	Lesser perceived frequency of positive interactions (T1) predicted increases in depressive symptoms (T2)
Feinstein et al. [56]	Depression	Perceived frequency of negative interactions	+	Depressive symptoms at T1 predicted more negative interactions at T2 with close friends and romantic partners
Feinstein et al. [56]	Depression	Perceived frequency of positive interactions	-	Depressive symptoms at T1 predicted less positive interactions at T2 with romantic partners
Frison et al. [81]	Depression	Negative peer experiences on SNSs	+	Cross-lagged analyses suggested a unidirectional relationship between depression (T1) and increases in negative Facebook experiences (T2; 6-month interval)
Landoll et al. [107]	Depression	Negative peer experiences on SNSs	+	

Study	Mental Health Focus	Key SNS Variables	Direct Associations	Additional Notes and Complex Relationships
Moberg & Anestis [108]	Depression	SNS interactions (very negative – very positive)	-	When controlling for depression, interaction ratings and thwarted belongingness had a significant negative relationship
Szwedo et al. [103]	Depression	Positive peer relationship quality	NS	
Szwedo et al. [103]	Depression	Negative peer relationship quality	-<br><br>+	Symptoms at age 13 (T1) predicted less deviancy talk from peers at age 20 (T2)<br><br>Symptoms at age 20 (T2) predicted more verbally aggressive comments from peers at T2
Feinstein et al. [56]	Anxiety	Perceived frequency of negative interactions	+	Global Anxiety symptoms at T1 did not predict interactions at T2, though cross-sectional associations were observed.
Feinstein et al. [56]	Anxiety	Perceived frequency of positive interactions	-	

Study	Mental Health Focus	Key SNS Variables	Direct Associations	Additional Notes and Complex Relationships
Feinstein et al. [56]	Social Anxiety	Perceived frequency of negative interactions	+	Social Anxiety symptoms at T1 did not predict interactions at T2, though cross-sectional associations were observed
Feinstein et al. [56]	Social Anxiety	Perceived frequency of positive interactions	-	Relationship is NS when only observing interactions with close friends
Hong et al. [109]	Social Anxiety (online)	Facebook continuance intention	-	Belief that virtual communities are dangerous (including the potential to encounter hostile/negative interactions) is positively associated with online and general social anxiety subsequently links to reduced continuance intention for using Facebook
Landoll et al. [107]	Social Anxiety	Negative peer experiences on SNSs	+	
Szwedo et al. [103]	Social Anxiety	Positive Peer Relationship Quality	+	Symptoms at age 20 (T2) predicted more supportive comments from peers at T2

	Study	Mental Health Focus	Key SNS Variables	Direct Associations	Additional Notes and Complex Relationships
	Szwedo et al. [103]	Social Anxiety	Negative Peer Relationship Quality	-	Symptoms at age 13 (T1) predicted fewer verbally aggressive comments from peers at age 20 (T2)
	Frison et al. [81]	*Life Satisfaction*	Negative peer experiences on SNSs	-	Cross-lagged analyses suggested a bidirectional relationship between life satisfaction (T1/T2) and negative Facebook experiences (T1/T2)
**Social Support**					
	Frison & Eggermont [110]	Depression	Perceived Social Support (via SNS)	-	
	Frison & Eggermont [110]	Depression	Social Support Seeking (via SNS)	+	Social support seeking through Facebook and its association with depressed mood was mediated by perceived social support (greater perceived social support decreased depressed mood).

Study	Mental Health Focus	Key SNS Variables	Direct Associations	Additional Notes and Complex Relationships
Frison et al. [81]	Depression	Perceived Friend Support (via SNS)-moderator<br><br>*(Negative Facebook experiences)*	-	Moderated by perceived friend support (low, medium, high), the association between negative Facebook experiences (T1) and depressed mood (T2) was significant only for low and medium levels.
McCloskey et al. [112]	Depression	Perceived social support (via SNS)	NS	
McCloskey et al. [112]	Depression	Emotional support (via SNS)	+	
McCloskey et al. [112]	Depression	Negative social support (via SNS)	+	
McCloskey et al. [112]	Depression	Instrumental support (via SNS)	NS	
Park et al. [93]	Depression	Perceived Social Support (via SNS)	-	
Park et al. [93]	Depression	Actual Social Support (via SNS)	NS	Significant positive relationship where the individual was disclosing negative sentiment and they were higher in depressive symptoms.

Study	Mental Health Focus	Key SNS Variables	Direct Associations	Additional Notes and Complex Relationships
Park et al. [93]	Depression	Discrepancy between actual and perceived support	+	Group differences -discrepancy is larger for individuals with MDD compared to non-depressed participants.
Wright et al. [79]	Depression	SNS Social support satisfaction	-	Path from higher CMC competence predicts more Facebook Social Support Satisfaction and subsequently lower depression scores. Motives to use Facebook for social integration and interpersonal communication also contributed to this model
Indian & Grieve [111]	Social Anxiety (high)	Perceived social support (via SNS)	+ (*subjective well-being*)	Controlled for offline social support<br><br>Group comparisons also revealed no significant differences in perceived Facebook social support between the low and high social anxiety groups
Indian & Grieve [111]	Social Anxiety (low)	Perceived social support (via SNS)	NS (*subjective well-being*)	

	Study	Mental Health Focus	Key SNS Variables	Direct Associations	Additional Notes and Complex Relationships
	Frison et al. [81]	*Life Satisfaction*	Perceived Friend Support (via SNS) - moderator<br><br>*(Negative Facebook experiences)*	+	Moderated by perceived friend support (low, medium, high), the association between negative Facebook experiences (T1) and life satisfaction (T2) was significant only for low and medium levels.
	McCloskey et al. [112]	*Quality of Life*	Perceived social support (via SNS)	NS	
	McCloskey et al. [112]	*Quality of Life*	Emotional support (via SNS)	-	Direct association only with psychological well-being of the WHOQOL-BREF domains
	McCloskey et al. [112]	*Quality of Life*	Negative social support (via SNS)	-	
	McCloskey et al. [112]	*Quality of Life*	Instrumental support (via SNS)	NS	
**Social Connectedness**					
	Grieve et al. [113]	Depression	Facebook social connectedness	-	
	Grieve et al. [113]	Anxiety	Facebook social connectedness	-	
	Grieve et al. [113]	*Life Satisfaction*	Facebook social connectedness	+	

	Study	Mental Health Focus	Key SNS Variables	Direct Associations	Additional Notes and Complex Relationships
**Social Comparison**					
	Appel et al. [116]	Depression	Envy	+	Significant interaction – envy was greater in response to viewing an attractive Facebook profile, with it being more so in a depressed group compared to a non-depressed group
	Tandoc et al. [78]	Depression	Envy	+	Facebook envy mediates the association between surveillance uses of Facebook and depression
	Lup et al. [68]	Depression	Downward Social Comparison on SNSs	-	Social comparison was an indirect mediator of the relationship between the frequency of Instagram use and depression. This pathway was additionally moderated by the proportion of strangers followed in the network.
	Steers et al. [77]	Depression	Downward Social Comparison on SNSs	+	Social comparison mediated the relationship between time spent on Facebook and depressive symptoms

Study	Mental Health Focus	Key SNS Variables	Direct Associations	Additional Notes and Complex Relationships
Steers et al. [77]	Depression	Non-directional Social Comparison on SNSs	+	Social comparison mediated the relationship between time spent on Facebook and depressive symptoms
Steers et al. [77]	Depression	Upward Social Comparison on SNSs	+	Social comparison mediated the relationship between time spent on Facebook and depressive symptoms
Feinstein et al. [115]	Depression	Social Comparison on SNSs	+	More negative social comparison on Facebook at T1 was associated with increases in depressive symptoms at T2 via increases in rumination (3-week interval).
Lee [114]	Depression	Social Comparison on SNSs	+	
Lee [114]	Anxiety	Social Comparison Frequency on SNSs	+	
Appel et al. [116]	*Self-Esteem*	Envy	-	
Lee [114]	*Self-Esteem*	Social Comparison on SNSs	-	

	Study	Mental Health Focus	Key SNS Variables	Direct Associations	Additional Notes and Complex Relationships
**Addictive or Problematic Use**					
	Andreassen et al. [117]	Depression	Addictive SNS use	-	Controlled for demographic characteristics, OCD, and ADHD
	Hanprathet et al. [119]	Depression	Addictive SNS use	+	
	Hong et al. [60]	Depression	Addictive SNS use	+	
	Koc & Gulyagci [62]	Depression	Addictive SNS use	+	
	Wegmann et al. [121]	Depression	Addictive SNS Use	+	Internet use expectancies and self-regulation mediate the relationship between SNS addiction and depression/social anxiety
	Blachnio et al. [118]	Depression	Facebook Intrusion (behavioural addiction)	+	
	Giota & Kleftaras [58]	Depression	Problematic SNS use	+	NS when accounting for personality and average daily SNS use

Study	Mental Health Focus	Key SNS Variables	Direct Associations	Additional Notes and Complex Relationships
Moreau et al. [120]	Depression	Problematic SNS use	+	Cluster analysis indicated that the ‘borderline group’ which had significantly higher social anxiety and depression scores than two other clusters, also had a higher level of problematic Facebook use
Andreassen et al. [117]	Anxiety	Addictive SNS use	+	Controlled for demographic characteristics, OCD, and ADHD
Hanprathet et al. [119]	Anxiety	Addictive SNS use	+	
Bodroža & Jovanović [106]	Social Anxiety	Addictive SNS use	+	
Wegmann et al. [121]	Social Anxiety	Addictive SNS Use	+	
Casale & Fioravanti [104]	Social Anxiety	Problematic SNS use	+	Males – The relationship between social anxiety and problematic SNS use is indirectly mediated by the *need for self-presentation*

	Study	Mental Health Focus	Key SNS Variables	Direct Associations	Additional Notes and Complex Relationships
	Lee-Won et al. [65]	Social Anxiety	Problematic SNS use	+	NSA moderated this relationship with it being more pronounced for those with high and medium NSA, and becoming NS for those low in NSA
	Moreau et al. [120]	Social Anxiety	Problematic SNS use	+	
	Hong et al. [60]	*Self Esteem*	Facebook Addiction	NS	
**Physiological Arousal and Facebook**					
	Raunch et al. [122]	Social Anxiety	Facebook exposure	+	High social anxiety had greater physiological arousal in a face-to-face encounter after prior exposure to a Facebook profile.

Note: "+" = significant positive relationship reported; "-" = significant negative relationship reported; "NS" = non-significant findings reported; SNS = Social Networking Site; CMC = Computer Mediated Communication; MDD = Major Depressive Disorder; NSA = Need for Social Reassurance; CES-D = Center for Epidemiologic Studies Depression Scale; PHQ-9 = Patient Health Questionnaire – 9; WHOQOL-BREF = WHO Quality of Life-BREF; BDI = Beck Depression Inventory; ESM = Experience Sampling Method; T0 = baseline; T1 = Time 1; T2 = Time 2

Frequency of SNS use refers to studies using a Likert type question with no defined time measurement (e.g. never – very frequently).
